# Comparison of monoplanar and polyaxial screw fixation systems in percutaneous intermediate fixation for thoracolumbar fractures

**DOI:** 10.1186/s12891-022-05129-8

**Published:** 2022-02-22

**Authors:** Liangliang Huang, Chengjie Xiong, Zhongyi Guo, Qiuyu Yu, Feng Xu, Hui Kang

**Affiliations:** Department of Orthopaedics, General Hospital of Central Theater Command, Wuhan, 430070 Hubei China

**Keywords:** Thoracolumbar fracture, Monoplanar pedicle screw, Polyaxial pedicle screw, Percutaneous intermediate fixation, Percutaneous pedicle screw fixation

## Abstract

**Background:**

The newly developed monoplanar pedicle screws (MPPSs) can mobile in axial plane but fixed in the sagittal plane, which holds potential to combine ease of rod placement with sagittal plane strength theoretically. So far, few clinical studies focused on the outcomes of MPPSs for treatment of thoracolumbar fractures (TLFs). The aim of this study was to compare the efficacy of MPPSs to polyaxial pedicle screws (PAPSs) in percutaneous intermediate fixation of TLFs.

**Methods:**

Seventy-eight patients who sustained TLFs without neurological deficits and underwent percutaneous intermediate fixation using MPPSs (40 patients) or PAPSs (38 patients) with a minimum 1-year follow-up were included in this study. The operation time, blood loss, local Cobb angle (LCA), vertebral wedge angle (VWA), anterior body height ratio (ABHR), visual analogue scale (VAS) and Oswestry Disability Index (ODI) were collected.

**Results:**

No significant differences were observed in baseline demographics, clinical characteristics, operation time or blood loss between the two groups (*P* > 0.05). The postoperative LCA, VWA and ABHR were significantly corrected compared to these parameters preoperatively in both groups (^#^*P* < 0.05). The postoperative LCA, VWA and ABHR in the MPPS group were significantly better corrected than those in the PAPS group (*P < 0.05). Furthermore, the correction loss of LCA, VWA and ABHR in the MPPS group was significantly lower than that in the PAPS group (*P < 0.05). However, no significant difference in VAS and ODI scores was observed between the two groups.

**Conclusions:**

MPPSs showed similar efficiency as PAPSs in percutaneous intermediate fixation surgical procedures. More importantly, MPPSs achieved better radiological performance than PAPSs in the correction of TLFs and the prevention of correction loss.

**Supplementary Information:**

The online version contains supplementary material available at 10.1186/s12891-022-05129-8.

## Background

Spine fractures are commonly observed in traffic accidents, height crashes and other high-energy injuries, and they account for 5% of all trauma patients [1]. Approximately 60–70% of all traumatic spinal fractures are thoracolumbar fractures (TLFs, from T11 to L2), which is ascribed to the special biomechanical characteristics of the thoracolumbar spine that translate from the rigid, kyphotic thoracic spine to the mobile, lordotic lumbar spine [2]. Surgical or conservative management of TLFs remains controversial, especially for patients without neurological deficits [3, 4].

In recent years, minimally invasive percutaneous pedicle screw fixation (PPSF) for TLFs has been growing in popularity [5–7]. The typical PPSF for TLFs is 4-screw fixation with pedicle screws introduced to one level above and below the injured vertebra. Recently, a series of studies have documented that percutaneous intermediate fixation (PIF), which adds two screws in the fractured vertebra, provides stronger fixation than traditional 4-screw fixation [2, 8, 9]. In addition, PIF is more effective in restoring and maintaining fractured vertebral height [2, 9–11]. The thoracolumbar spine is the junctional area from a narrow thoracic spine to a wider lumbar spine; it requires inserting screws with high alignment, and slight deviation of the ipsilateral three screws might make it difficult to insert the longitudinal rod, especially using fixed-axis pedicle screws in PIF (6-screw fixation) [12, 13]. The polyaxial pedicle screw (PAPS) increases the degrees of freedom at the screw-rod interface and provides greater ease for rod insertion, making PAPSs more favourable than traditional fixed-axis pedicle screws in minimally invasive surgeries [12, 13]. PAPSs show less stiffness in the sagittal plane and inferior fracture reduction compared to fixed-axis pedicle screws in TLFs [12, 14].

Recently, a novel type of pedicle screw, monoplanar pedicle screw (MPPS), has been introduced. MPPS is designed to be mobile in the axial plane but fixed in the sagittal plane. Thus, MPPS behaves as a fixed-axis pedicle screw in the sagittal plane and a PAPS in the axial plane. A series of recent biomechanical studies have demonstrated that MPPSs significantly increase stiffness in the sagittal plane compared to PAPSs [15–17]. Theoretically, MPPSs may combine the ease of rod insertion with sagittal plane strength.

To our knowledge, few clinical studies have focused on the outcomes of MPPSs for the treatment of TLFs. The objectives of this study were to compare the efficacy of MPPSs to PAPSs in PIF for TLFs without neurological deficits.

## Methods

### Patient population

A retrospective study was adopted to review patients who underwent PIF surgery using MPPSs or PAPSs from January 2017 to May 2020. A total of 78 patients were enrolled in this study. The patients were divided into two groups: those treated with the MPPS system were included in the MPPS group; those treated with the PAPS system were included in the PAPS group. All protocols were approved by the Ethics Committee of General Hospital of Central Theater Command (approval number: [2021]040) and were performed in compliance with the Helsinki Declaration.

#### Inclusion and exclusion criteria

The inclusion criteria for patients were as follows: traumatic fracture of T11-L2; type A3 and A4 with or without type B2 in AO classification of spinal fracture [18]; without neurologic deficit; age 18 to 60 years; less than 2 weeks between trauma and surgery; and followed up for more than 12 months. Patients with the following criteria were excluded: fractures of more than 2 vertebrae; presence of nerve injury symptoms; pregnancy; pathologic or osteoporotic fracture; bilateral pedicle fracture; and a history of previous spinal surgery. The general information of the patients is summarized in Table 1.

**Table 1:** Baseline demographic and clinical characteristics of patients

Paramater	MPPS (*n* = 40)	PAPS (*n* = 38)	*P* value
Age (years)	41.8 ± 11.1 (19.4–60.0)	42.1 ± 10.4 (19.1–58.5)	0.930
Gender			
Males	29	30	0.507
Females	11	8	
Fracture mechanism			
Traffic accident	15	15	0.703
Fall from height	19	15	
others	6	8	
Fracture level			
T11	2	4	0.682
T12	18	13	
L1	14	14	
L2	6	7	
Fracture type			
Type A3	25	26	0.876
Type A4	4	2	
TypeB2 A3	9	9	
Type B2A4	2	1	
TLICS	4.0 ± 0.7 (3–5)	4.0 ± 0.7 (3–5)	0.756
Follow-up (months)	15.9 ± 3.2 (12.2–24.8)	16.6 ± 3.9 (12.5–26.3)	0.358

*MPPS* Monoplanar pedicle screw, *PAPS* Polyaxial pedicle screw

##### Surgical procedures

**Fig. 1:**
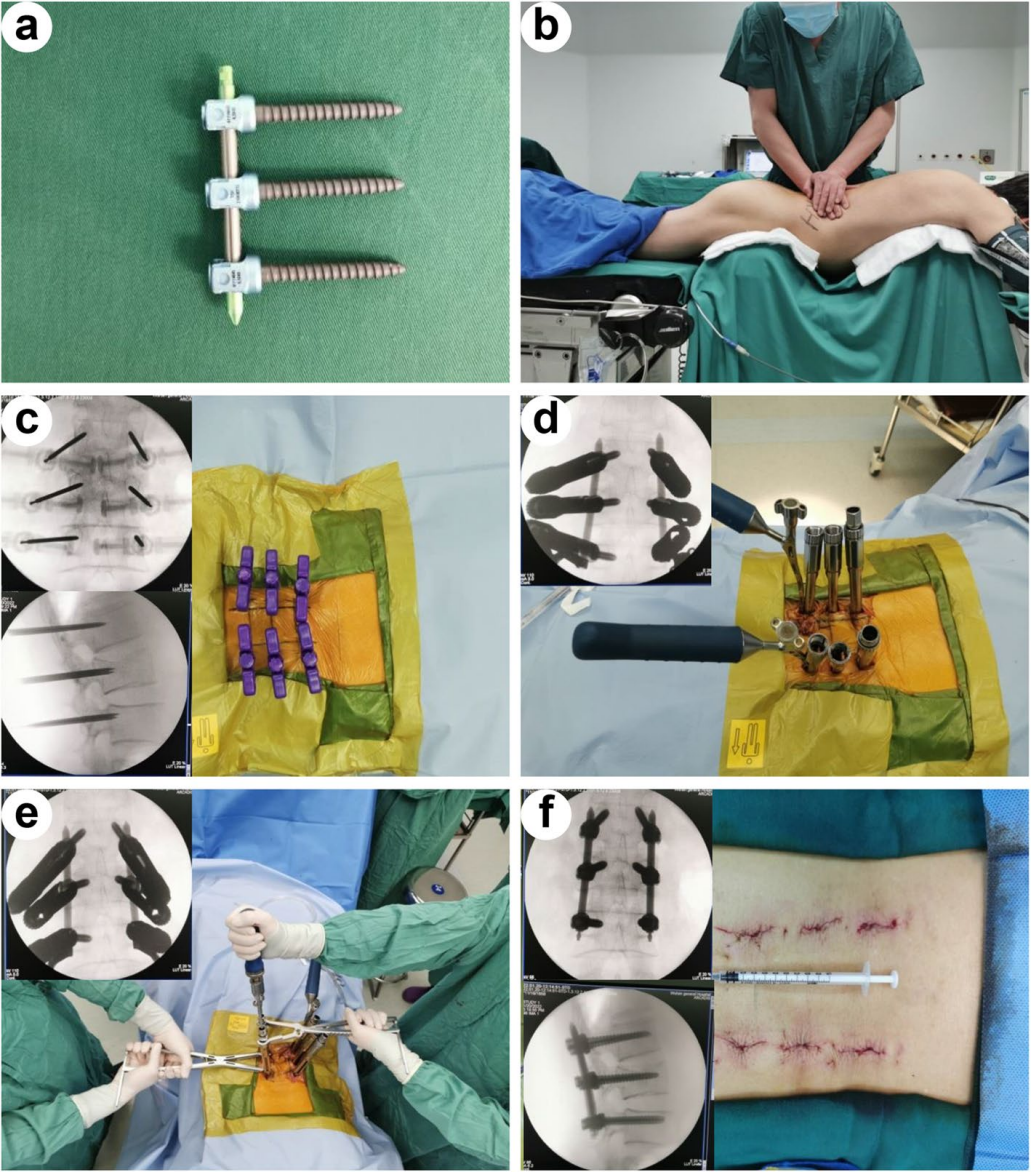
Representative images of surgical procedures of a 57-year-old male patient in the MPPS group. **a** Representative image of MPPSs. **b** Application of manual forces on the fractured vertebra to correct kyphosis. **c** Six Jamshidi needles were inserted into the pedicles and vertebral bodies. (d) Six MPPSs were implanted into the thoracolumbar region and two rods with appropriate length and bending were inserted. **e** TLF was further corrected by applying a hyperlordosing force through the posterior elements before tightening the screws. **f** The location of internal fixation and correction of kyphosis were confirmed, and the incisions were closed

All surgeries were performed by fully qualified spine surgeons as reported previously (Fig. 1) [19–21]. Under general endotracheal anaesthesia, all patients were operated in the prone position on a Jackson operating table with chest and pelvis supported by a pad and abdomen suspended. The location of the pedicles of the fractured level, and one level above and below the injured vertebra were marked according to the posteroanterior fluoroscopy. One surgeon applied manual forces on the fractured vertebra by his hands under intermittent C-arm fluoroscopic guidance (Fig. 1b). Continuous neuromonitoring was utilized to monitor neural function. Skin incisions were made 1.5-cm lateral from the marks of those pedicles. Each pedicle was cannulated by a Jamshidi needle with proper direction and depth. A guidewire was placed into the vertebral body through the needle. The pedicle screw was inserted into the pedicle and vertebral body along the wire. There were six percutaneous pedicle screws (one level above and below the injured vertebra as well as the fractured level) implanted into each patient’s thoracolumbar region in both MPPS group and PAPS group [22]. After implantation of all six screws, rods with appropriate length and bending were inserted (Fig. 1d). The TLF was further corrected by applying a hyperlordosing force through the posterior elements before tightening the screws (Fig. 1e). Posteroanterior fluoroscopy and lateral fluoroscopy were conducted to verify the reduction effect (Fig. 1f). The duration of operation time and the amount of blood loss were recorded. Postoperative management was performed as reported previously [23, 24]. All patients were encouraged to partake in ambulatory activities while wearing a brace 3 days after surgery.

##### Clinical evaluation

Low back pain was evaluated by the VAS (0–10 scale). The functional outcomes were assessed by ODI as described previously [25]. The VAS score and ODI were assessed preoperatively, 5 days postoperation, 1 month postoperation and at the last follow-up.

##### Radiological evaluation

**Fig. 2:**
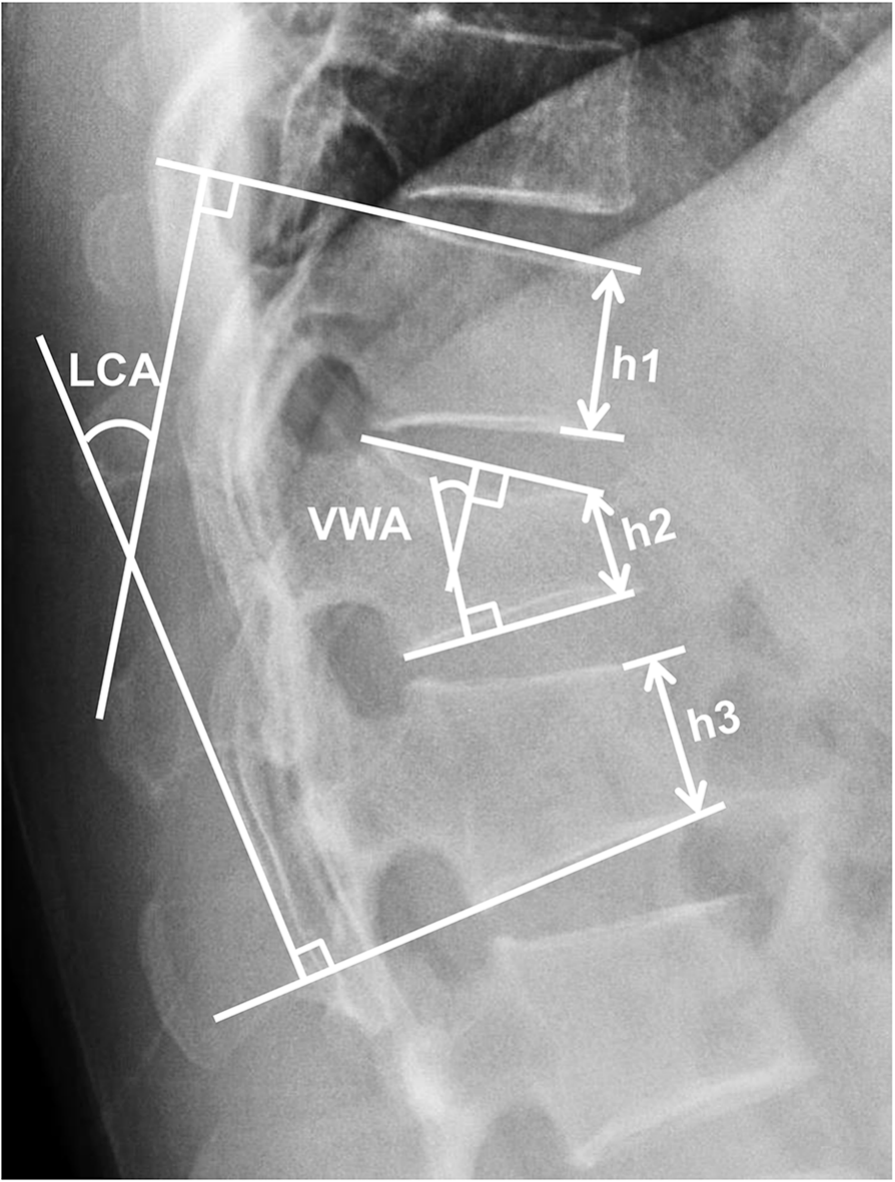
Measurement of radiological parameters. LCA, local Cobb angle; VWA, vertebral wedge angle; ABHR, anterior body height ratio (ABHR = h2 / [(h1 + h3) / 2] × 100%)

Thoracolumbar anterior-posterior and lateral X-rays in addition to thoracolumbar computed tomography (CT) and magnetic resonance imaging (MRI) were obtained before the operation to evaluate the fracture. The LCA, VWA and ABHR (Fig. 2) were evaluated by lateral X-rays. The LCA was measured between the superior endplate of the vertebra above the injured vertebra and inferior endplate of the vertebra below the injured vertebra. VWA was defined as the Cobb angle of the fractured vertebra, and ABHR was defined as the percentage of the anterior body high of fractured vertebra to the mean value of the adjacent vertebrae. All data were measured by two independent observers who were blinded to the group assignment.

##### Statistical analyses

Continuous variables are presented as the mean ± SD and were evaluated by Student’s t test. Categorical variables are presented as numbers and were compared utilizing the chi-square test or Fisher’s exact test. A statistical significance level of *P* < 0.05 was applied. Statistical analyses were conducted by SPSS 23.0 (IBM, New York, USA).

## Results

In total, 78 patients with traumatic TLFs were included as follows: 40 patients received MPPSs, and 38 patients received PAPSs. The demographic data and clinical characteristics of all patients are shown in Table 1. There was no significant difference in age, sex, fracture mechanism, fracture level or AO classification between the two groups (*P* > 0.05). The average TLICS was 4.0 ± 0.7 (3–5) in both groups (*P* > 0.05). The average time of follow-up was 15.9 ± 3.2 (12.2–24.8) months for the MPPS group and 16.6 ± 3.9 (12.5–26.3) months for the PAPS group (*P* > 0.05). The mean operation time was 80.5 ± 15.4 (64.3–119.5) min in the MPPS group and 78.3 ± 16.2 (63.8–125.5) min in the PAPS group (*P* > 0.05). The blood loss was 87.5 ± 37.1 (45–155) ml in the MPPS group and 80.3 ± 34.0 (40–165) ml in the PAPS group (*P* > 0.05).

**Fig. 3:**
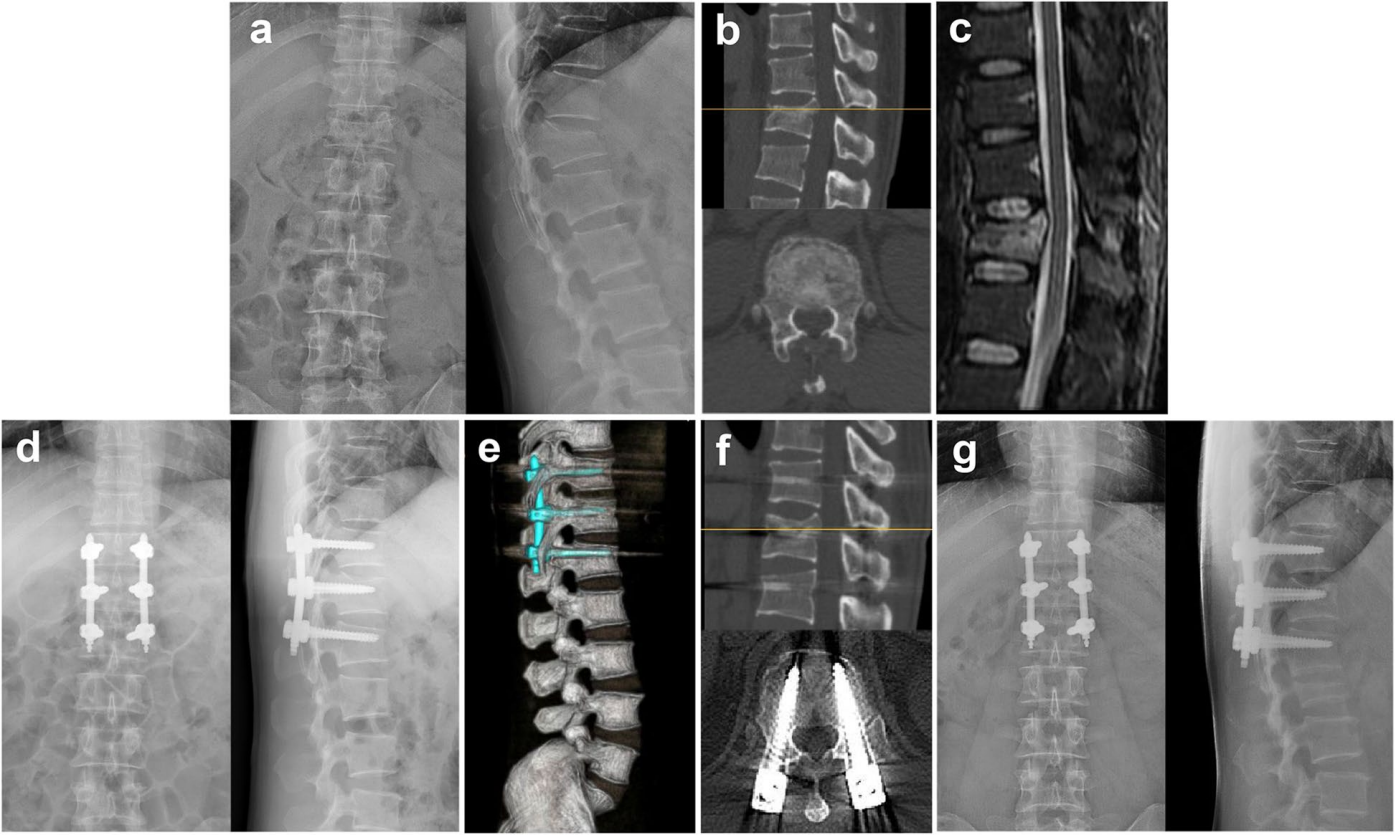
A 39-year-old male patient from the MPPS group. Preoperative X-ray (a) and CT (b) demonstrated T12 AO type B2A3 fracture without apparent neurological deficit. There was marked widening of the interspinous distance between T11 and T12. MRI (c) showed fresh fracture of the T12 vertebral and interspinous ligament injury. Postoperative X-ray (d) and CT (e - f) showed satisfactory traumatic kyphosis correction and vertebral height restoration. X-ray image (g) at 15 months following surgery

**Fig. 4:**
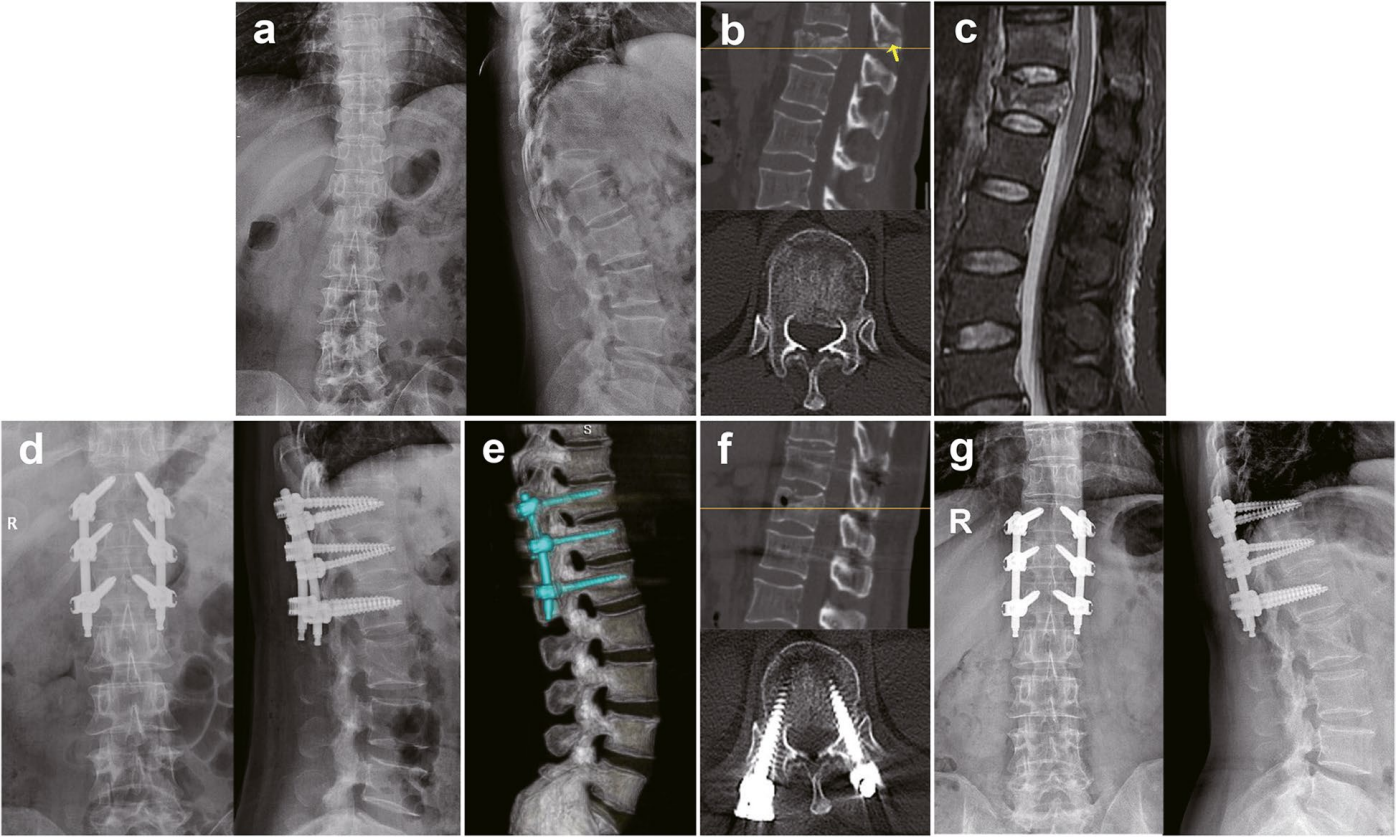
A 45-year-old female patient from the PAPS group. Preoperative X-ray (**a**) and CT (**b**) demonstrated a T12 AO type B2A3 fracture without apparent neurological deficits. The arrow indicates the fracture of the T11 spinous process. MRI (**c**) showed fresh fracture of the T12 vertebral and T11 spinous processes. Postoperative X-ray (**d**) and CT (**e** - **f**) showed satisfactory traumatic kyphosis correction and vertebral height restoration. X-ray image (**g**) at 14 months following surgery

As shown in Table 2, there was no significant difference in the preoperative LCA, VWA or ABHR between the MPPS and PAPS groups (*P* > 0.05). As expected, the postoperative LCA, VWA and ABHR were significantly corrected compared to these parameters preoperatively in both groups (Table 2, Fig. 3 and Fig. 4, ^#^*P* < 0.05). The postoperative LCA in the MPPS group (6.0° ± 5.1°) was significantly lower than that in the PAPS group (9.2° ± 4.7°, *P < 0.05). Similarly, the postoperative VWA and ABHR in the MPPS group were also significantly better corrected than those in the PAPS group (*P < 0.05). Both the MPPS and PAPS groups showed correction loss with time. Importantly, the LCA, VWA and ABHR in the MPPS group at the last follow-up remained significantly better than those in the PAPS group (*P < 0.05). In addition, significant differences were also found in the correction loss of LCA, VWA and ABHR between the two groups (Table 2, *P < 0.05).

**Table 2:** Summary of radiographic measurements

Paramater	MPPS (*n* = 40)	PAPS (*n* = 38)	*P* value
Local Cobb angle (LCA) (°)			
Preoperative LCA	20.4 ± 7.3	21.6 ± 8.0	0.509
Postoperative LCA	6.0 ± 5.1^#^	9.2 ± 4.7^#^	0.005*
LCA at Last follow-up	7.8 ± 5.8	11.9 ± 5.4	0.002*
Correction loss	1.8 ± 1.2	2.7 ± 1.4	0.002*
Vertebral wedge angle (VWA) (°)			
Preoperative VWA	18.7 ± 5.6	19.1 ± 6.4	0.771
Postoperative VWA	5.8 ± 3.1^#^	8.2 ± 4.5^#^	0.005*
VWA at Last follow-up	7.1 ± 3.5	10.2 ± 4.9	0.002*
Correction loss	1.3 ± 0.9	2.0 ± 1.0	0.003*
Anterior body height ratio (ABHR)(%)			
Preoperative ABHR	65.1 ± 9.2	65.3 ± 9.3	0.938
Postoperative ABHR	94.8 ± 6.5^#^	91.1 ± 6.7^#^	0.017*
ABHR at Last follow-up	92.5 ± 6.2	87.7 ± 8.0	0.004*
Correction loss	2.3 ± 1.4	3.4 ± 2.0	0.003*

^*^P < 0.05 compared between the MPPS and PAPS groups; ^*#*^*P* < 0.05 compared between the postoperative and preoperative radiographic results

The VAS for back pain and ODI scores were significantly improved following surgery in both groups (Table 3, ^#^*P* < 0.05) and gradually improved with time. However, no significant differences were found in VAS and ODI between the two groups at any time point (Table 3, *P* > 0.05). There were no major vascular injuries or neurologic complications. No patient required revision for correction loss or instrumentation failure at the last follow-up.

**Table 3:** Clinical outcomes between the two groups

Paramater	MPPS (*n* = 40)	PAPS (*n* = 38)	*P* value
VAS			
Pre-operation	7.8 ± 1.4	7.7 ± 1.5	0.909
5 day postoperation	2.5 ± 0.7^*#*^	2.7 ± 1.2^*#*^	0.277
1 month postoperation	1.4 ± 0.9	1.7 ± 0.9	0.079
Last follow-up	1.1 ± 0.9	1.2 ± 0.8	0.670
ODI			
Preoperation	87.0% ± 11.6%	889.3% ± 10.2%	0.363
5 day postoperation	60.0% ± 6.4%^*#*^	62.0% ± 6.4%^*#*^	0.159
1 month postoperation	33.1% ± 4.2%	34.5% ± 5.4%	0.183
Last followup	6.8% ± 4.0%	7.4% ± 5.3%	0.529

*VAS* Visual analogue score, *ODI* Oswestry Disability Index

^#^*P* < 0.05 compared between the 5 day postoperation and preoperation clinical results

## Discussion

Traumatic TLFs are commonly observed in traffic accidents, height crashes and other high-energy injury situations [1]. Open surgery has been recommended for patients with neurological deficits and unstable TLFs to decompress the nerve and stabilize the spine. For patients without neurological deficits, decompression is not required, and the treatment focuses on recovering the height of the fractured vertebra, restoring the stability of the spine and avoiding complications due to posttraumatic kyphosis and prolonged bed rest [26]. Minimally invasive PPSF exhibits better fracture reduction and long-term clinical outcomes than nonoperative treatment, and it provides three-column fixation similar to open surgery and shows less soft tissue injury, lower infection risk, less postoperative pain and shorter rehabilitation time than traditional open surgery [5–7]. Therefore, PPSF has been growing in popularity for the treatment of TLFs [7]. With the development of PPSF, more recent studies have demonstrated that PIF, which adds two screws to the fractured vertebra, is more effective in restoring and maintaining fractured vertebral height than the classic 4-screw PPSF [2]. In the present study, all the patients received PIF using six pedicle screws. Our results showed that the LCA, VWA and ABHR were significantly corrected by surgery in both groups. Our results provided further evidence that PIF effectively recovers the height of fractured vertebrae and corrects kyphosis.

In PIF surgery, high alignment of the ipsilateral three screws is required for successful installation of the longitudinal connecting rod, especially using fixed-axis pedicle screws. Deviation in the position of screw placement may increase the difficulty of surgery and prolong the operation time [12, 13]. PAPSs increase the degrees of freedom at the screw-rod interface and provide greater ease for rod insertion. However, it has been reported that fixed-axis pedicle screws provide better correction of deformities than PAPSs [14]. In an effort to combine the relative advantages of fixed-axis pedicle screws and PAPSs, MPPSs were developed. MPPSs are mobile in the axial plane (see Additional file 1), which may facilitate rod insertion and improve surgical efficiency. In the present study, the mean operation time and blood loss showed no significant difference between the MPPS group and the PAPS group (*P* > 0.05). These results indicated that the operating efficiencies of MPPSs and PAPSs were comparable.

MPPSs are rigid in the sagittal plane and behave as fixed-axis screws in the sagittal plane (see Additional file 1) [16], potentially achieving better fracture reduction and kyphotic angle correction similar to fixed-axis screws. In line with this hypothesis, our results demonstrated that the LCA, VWA, and ABHR were better corrected by MPPSs than PAPSs. In addition, MPPSs showed less correction loss than PAPSs with prolonged follow-up.

In the present study, statistically significant differences were not observed in the VAS and ODI scores between the two groups. The following reasons might account for this lack of statistical difference: 1) both MPPS and PAPS fixation procedures are minimally invasive procedures that cause little damage to paraspinal soft tissues [6, 26]; and 2) one of the most important reasons for functional defects after TLF is pain caused by the jiggle of the fractured vertebra under loading-bearing stress, buckling stress and rotation stress, and both MPPS and PAPS fixation provide sufficient strength to stabilize the fractured vertebra and minimize the pain caused by the jiggle of the fractured vertebra. Further studies with long-term follow-up after the removal of instrumentations might clarify whether the promising radiological results in the MPPS group translate to superior functional outcomes.

There were several limitations to this study. First, this was a retrospective study without randomization, potentially resulting in selection bias. A randomized, prospective study is warranted to further confirm these findings. Second, this was a one-center study, and the sample size remained small. Third, the follow-up time was relatively short without removal of instrumentations. It would be interesting to investigate the long-term functional outcomes, correction loss and adjacent segment degeneration after the removal of instrumentations in future studies.

## Conclusions

In summary, MPPSs and PAPSs showed similar operation times, blood loss and clinical outcomes in PIF of thoracolumbar fractures. The LCA, VWA, and ABHR were better corrected by MPPSs than PAPSs. In addition, MPPSs showed less correction loss than PAPSs with prolonged follow-up. These results highlighted that MPPS fixation system was a highly efficient fixation system as PAPS in PIF surgical procedures and MPPSs achieved better radiological performance than PAPSs in the correction of TLFs and the prevention of correction loss.

## Supplementary Information

**Additional file 1.** MPPS mobiles in the axial plane but fixes in the sagittal plane. Thus, MPPS behaves as a fixed-axis pedicle screw in the sagittal plane and a PAPS in the axial plane.

## Data Availability

The datasets analysed during the current study are available from the corresponding author on reasonable request.

## Abbreviations

ABHR: Anterior body height ratio
CT: Computed tomography
LCA: Local Cobb angle
MRI: Magnetic resonance imaging
MPPS: Monoplanar pedicle screws
ODI: Oswestry Disability Index
PAPS: Polyaxial pedicle screws
PIF: Percutaneous intermediate fixation
TLF: Thoracolumbar fractures
VAS: Visual analogue scale
VWA: Vertebral wedge angle
